# 3D Optical Vortex Trapping of Plasmonic Nanostructure

**DOI:** 10.1038/s41598-018-30948-y

**Published:** 2018-08-23

**Authors:** Jiunn-Woei Liaw, Chiao-Wei Chien, Kun-Chi Liu, Yun-Cheng Ku, Mao-Kuen Kuo

**Affiliations:** 1 Department of Mechanical Engineering, Chang Gung University, Taoyuan, Taiwan; 2 Institute for Radiological Research, Chang Gung University and Chang Gung Memorial Hospital, Taoyuan, Taiwan; 3 Center for Advanced Molecular Imaging and Translation, Chang Gung Memorial Hospital, Linkou, Taiwan; 4 Department of Mechanical Engineering, Ming Chi University of Technology, New Taipei City, Taiwan; 5 Institute of Applied Mechanics, National Taiwan University, Taipei, Taiwan

## Abstract

3D optical vortex trapping upon a polystyrene nanoparticle (NP) by a 1D gold dimer array is studied theoretically. The optical force field shows that the trapping mode can be contact or non-contact. For the former, the NP is attracted toward a corresponding dimer. For the latter, it is trapped toward a stagnation point of zero force with a 3D spiral trajectory, revealing optical vortex. Additionally the optical torque causes the NP to transversely spin, even though the system is irradiated by a linearly polarized light. The transverse spin-orbit interaction is manifested from the opposite helicities of the spin and spiral orbit. Along with the growth and decline of optical vortices the trapped NP performs a step-like motion, as the array continuously moves. Our results, in agreement with the previous experiment, identify the role of optical vortex in the near-field trapping of plasmonic nanostructure.

## Introduction

The optical-tweezers trapping on a microparticle or nanoparticle (NP) has been extensively studied for more than two decades^1–8^. Recently, using tightly focused circularly polarized (CP) Gaussian beam or Laguerre-Gauss (LG) beam to induce an optical vortex trapping associated with longitudinal or transverse spin angular momentum (SAM) and orbital angular momentum (OAM) has attracted considerable attentions^6–14^. On the other hand, using plasmonic nanostructure to induce optical vortex with sub-wavelength confinement has also been proposed^15–26^. Due to the collective motion of free electrons in metallic nanostructures corresponding to incident light, the plasmon-enhanced near fields can assist a variety of applications within the plasmon band of the nanostructures. For example, ref.^15^ has shown the plasmon-enhanced optical trapping of gold dimer arrays on a polystyrene NP in oil using 1064-nm laser beam; the suppression of NP’s Brownian motion by this plasmonic nanostructure is superior to the conventional optical tweezers. In addition, the NP was observed to move with a step-like manner from one stable trapping point generated in the near field of a corresponding dimer to the next one as the focused laser beam moves relative to and along the dimer array^15^. Because the plasmon behavior is broadband, near infrared (NIR) laser beams are preferred to be used for generating a locally enhanced near field but with less heating. In another research, the optical vortex trapping on polystyrene NP in water by diabolo nanoantenna array was found; a low-power laser of wavelength near the surface plasmon resonance of the gold nanoantenna was used^16^. From the optical force map, the NP is strongly trapped at two optical vortices on the surface of a thin and flat nanoantenna; the optical potential well identifies the trapping points^16^. Based on these studies, we raise a question: what is the role of 3D optical vortex playing for plasmon-enhanced trapping?

On the other hand, the light-matter interaction reveals the optical spin Hall effect, which is associated with the spin-orbit interaction (SOI) or the spin-momentum locking, linking the linear and spin/orbital angular momenta, have drawn lots of attentions recently^27–40^. The SOI involves the transportation of OAM with SAM of the longitudinal and transverse components^31–34^. For example, the transverse SOI was identified via the optomechanical responses of a metallic NP in an evanescent or surface plasmon polarition field^30–32,36^. In particular, as the optical fields are spatially nonhomogeneous in the near fields of plasmonic nanostructures the SOI becomes significant^37^. Of interest is that the induced transverse angular momentum of light possesses the photonic wheels behavior^41,42^. In addition, the concept of vortex nanogear transmission has also been proposed, which involves strong coupling SOIs of plasmonic dimer array^43,44^. Besides, the role of non-conservative optical force (curl force) on optical vortex was studied^45–47^.

**Figure 1:**
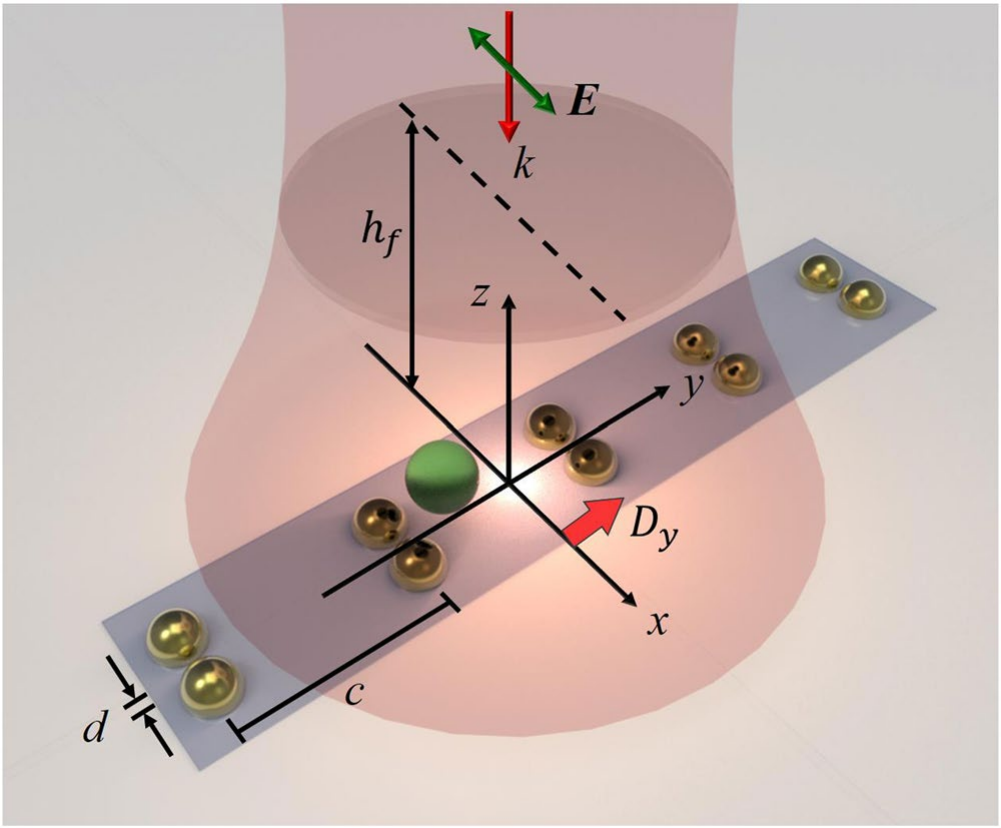
Configuration of 1D gold dimer array trapping a NP as irradiated by a normally incident LP Gaussian beam. The gap of dimer, lattice constant and displacement along the *y* direction of array are denoted by *d, c* and *D*_*y*_. The height of the focal plane and waist of the Gaussian beam with *x*-polarization are denoted by *h*_*f*_ and *w*_*0*_. The orientation of dimer is parallel to the *x* direction.

In this paper, we theoretically study the plasmon-enhanced trapping of a 1D gold dimer array induced by a normally incident linearly polarized (LP) NIR Gaussian beam to identify the role of 3D optical vortex playing^15^. The configuration of the structure is shown in Fig. 1, where the polarization of light is along *x* direction. The configuration of dimer is simplified by a pair of adjacent gold NPs. Because the refractive index of glass is the same with that of oil, which is 1.5, in the following simulation we assume that the dimer array is in immersion oil without considering the substrate effect. Throughout this paper, the waist of an *x*-polarized Gaussian beam of *λ* = 1064 *nm* is *w*_0_ = 500 *nm*. The refractive index of the polystyrene NP with a radius *a* = 100 *nm* is 1.6. The diameter of each gold NP of dimer is 130 *nm* with a gap *d* of 30 *nm*, and the lattice constant between adjacent dimers is 500 *nm*^15^. In the experiment of ref.^15^, the focal plane is at the height of *h*_*f*_ = 700 nm from the substrate, which is placed at the cross section of dimer array (*z* = 0). The displacement between the central dimer and the Gaussian beam is denoted by *D*_*y*_. The fluence of Gaussian beam at the center of focal plane is 25 MW/cm^2^. The multiple multipole (MMP) method, which is a semi-analytical one, will be used to simulate the electromagnetic (EM) field^48,49^. Subsequently, we will employ the Maxwell’s stress tensor, exhibiting the linear momentum flux of EM field, to calculate the optical force and torque upon the polystyrene NP exerted by EM field^50^. Through the optomechanical responses, the transverse SOI will be investigated. The transverse direction is defined to be perpendicular to the propagation direction of Poynting vector, which is the longitudinal direction. In this paper, we study the optomechanics of a finite-size Mie NP without using the Rayleigh dipole approximation^36,45^.

If we neglect the gravity and buoyancy forces as well as Brownian motion, the speed of a moving NP driven by the optical force will reach the terminal speed as the optical driving force **F** is balanced by the reactive drag force **F**_*s*_ from viscous fluid. According to the Stokes law, the drag force is ${{\bf{F}}}_{s}=-\,6\pi a\mu \,{\bf{v}}$, where *μ* is the dynamic viscosity of fluid and *a* the radius of NP. Consequently, the terminal velocity can be expressed by ${{\bf{v}}}_{T}={\bf{F}}/(6\pi a\mu )$. Therefore we can draw the velocity streamline of a NP by utilizing the optical force field, which is the trajectory of the NP’s quasi-static motion. In this paper, we only consider the quasi-static motion of NP. For a real motion, the inertia term of NP needs to be considered for calculating dynamic equation to trace its trajectory^9,51^. The velocity of the transient motion of a moving NP in a viscous medium is expressed by ${\rm{v}}={{\rm{v}}}_{T}(1-{e}^{-t/{t}_{0}})$, where the terminal speed is ${{\rm{v}}}_{T}=F/(6\pi a\mu )$. The time constant *t*_0_ for the transient motion to reach the terminal speed is ${t}_{0}=2{\rho }_{p}{a}^{2}/9\mu$, where *ρ*_*p*_ is the density of NP. For a polystyrene NP of *a* = 100 nm in oil, the time constant is about 10^−10^ *sec*, which is very short. Hence the assumption of quasi-static motion for a moving NP is reasonable; ${\rm{v}}={{\rm{v}}}_{T}$. On the other hand, the Stokes law illustrates that the viscous resistant torque on a spinning sphere in a viscous fluid is proportional to the spin speed **Ω**, ${{\bf{M}}}_{s}=-\,8\pi {a}^{3}\mu \,{\boldsymbol{\Omega }}$^49^. As the optical spin torque **M**_*c*_ is balanced by the viscous resistant torque **M**_*s*_, the terminal spin speed of a spinning NP is reached, expressed by ${{\boldsymbol{\Omega }}}_{T}={\bf{M}}{}_{c}/(8\pi {a}^{3}\mu )$. Hence the optical spin torque can be used to quantitatively express the quasi-static spinning of a NP.

## Results

### Optical vortices with stagnation point

**Figure 2:**
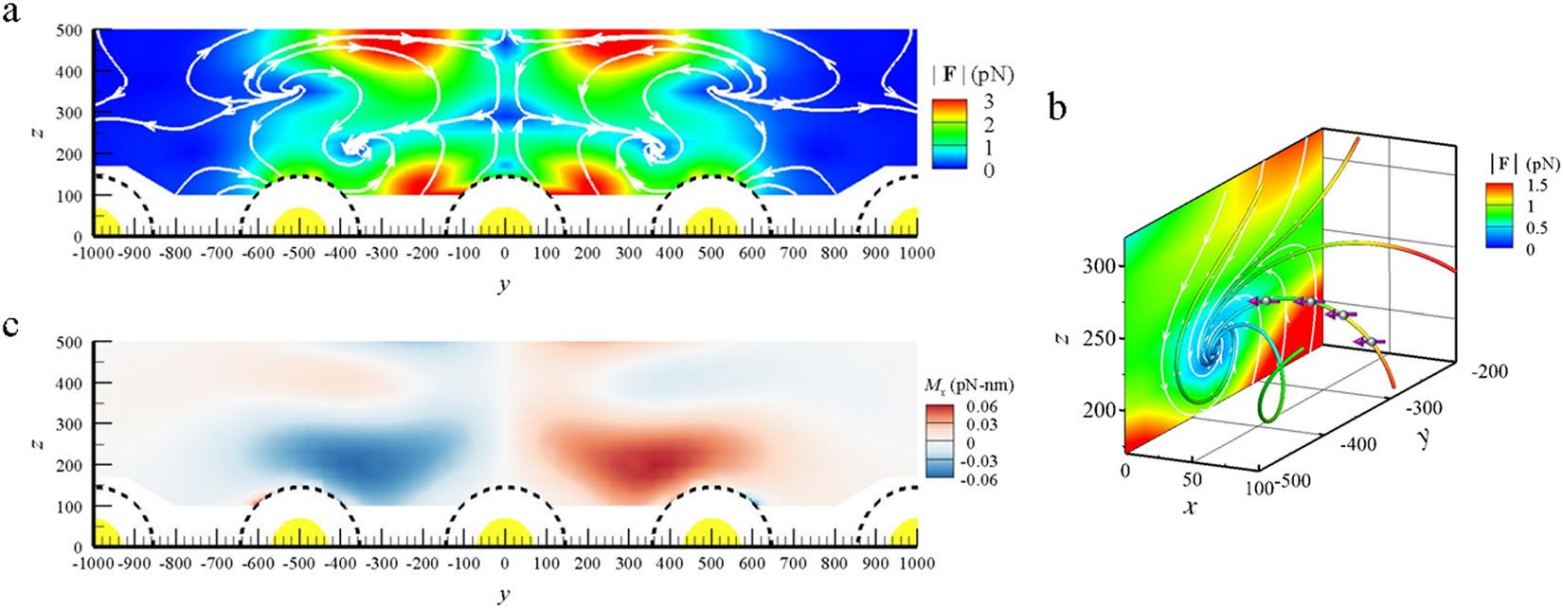
Optical vortex manifested by streamline map of optical force field. (**a**) The streamline map of the optical force field (*F*_*y*_, *F*_*z*_) for a polystyrene NP centered at different locations (*y*, *z*) in the *yz* plane of *x* = 0. The arrow and color of the optical force field represent the direction and amplitude, respectively. The dash line is the limit of the center of the NP with a radius of 100 *nm*. This map demonstrates that there are contact and non-contract modes for the optical trapping. For non-contract mode, two optical vortices are observed with stagnation points at ${{\bf{x}}}_{s}=(0,\,\pm \,361\,nm,\,207\,nm)$. (**b**) The 3D streamline map of the optical force field at the left-hand side optical vortex. (**c**) The map of the transverse optical spin torque field, *M*_*cx*_. The opposite helicities of the spin and spiral orbit of the probing NP indicate the transverse SOI. (*d* = 30 *nm*, *D*_*y*_ = 0 *nm*, *h*_*f*_ = 700 *nm*).

At first we assume that the optical axis of Gaussian beam normally impinges on the center dimer, i.e. *D*_*y*_ = 0 *nm*. Figure 2a shows the streamline map of the optical force field (*F*_*y*_, *F*_*z*_) for the NP centered at different locations in the *yz* plane of *x* = 0, where *F*_*x*_ = 0 due to the geometric symmetry. The arrow and color of the vector field represent the direction and amplitude of the optical force, respectively. Our numerical result demonstrates that there are contact and non-contract modes for the optical trapping. For the contact mode, the NP is attracted to attach to a dimer on the left-hand or right-hand side of Gaussian beam; e.g. the contact zone of the left-hand side dimer is on its high outside. For the non-contract mode, the NP is stably trapped at a stagnation point in an optical vortex by the left-hand or right-hand side dimer, rather than the central dimer. The two stagnation points are at ${{\bf{x}}}_{s}=(0,\,\pm \,361\,nm,\,207\,nm)$ in the near fields of both dimers; each optical vortex is near the high inside of the corresponding dimer. The two intact vortices are with opposite helicities. If the NP is placed away from the plane of *x* = 0 initially, the induced *F*_*x*_ will push it to this plane. Meanwhile, the other components of optical force (*F*_*y*_ and *F*_*z*_) drive the NP toward the stagnation point; a stagnation point performs like a sink. Figure 2b shows the 3D streamline plot of the left-hand side optical vortex. These typical 3D spiral trajectories (orbital motion combining with a centripetal motion) of the NP’s center toward the stagnation point manifest the 3D optical vortex caused by the optical force. The orbital motion is driven by ${{\bf{r}}}_{c}\times {\bf{F}}/\Vert {{\bf{r}}}_{c}\Vert$, and the centripetal motion by −${{\bf{r}}}_{c}\cdot {\bf{F}}/\Vert {{\bf{r}}}_{c}\Vert$. Here ${{\bf{r}}}_{c}$ is the relative position vector of NP’s center at ${{\bf{x}}}_{c}$ with respect to the stagnation point at ${{\bf{x}}}_{s}$; ${{\bf{r}}}_{c}={{\bf{x}}}_{c}-{{\bf{x}}}_{s}$. Once the NP is at the plane of *x* = 0, *F*_*x*_ vanishes. The 2D streamline map of *x* = 0 is also plotted in Fig. 2b. Since the radii of polystyrene NP and gold NP of dimer are 100 *nm* and 65 *nm*, respectively, the locations of these stagnation points are within the near field of dimer. The shortest distance between the surfaces of the polystyrene NP and either gold NP of the dimer is about 97 nm if the polystyrene NP is trapped at the stagnation point. Because the densities of polystyrene NP and oil are 1.04 g/cm³ and 0.923 g/cm³, respectively, the polystyrene NP is not supposed to float in oil statically. However, our result indicates that the NP can be trapped by the optical vortex to float without contacting with the dimer array. In addition, these optical vortices are highly localized; the characteristic length of the stagnation zone in the optical vortex is less than 100 *nm*, much smaller than the wavelength of light in oil (say 709 *nm*). The numerical results also indicate that the non-contact mode dominates the trapping over the contact one. Additionally, we found that the stagnation point performs as a sink, always accompanied by another source in its vicinity which is a forbidden zone for the probing NP to approach^46^.

### Transverse SOI

The spatial distribution of the *x*-component optical spin torque *M*_*cx*_ upon the dielectric NP with different locations in the *yz* plane of *x* = 0 is also plotted in Fig. 2c for *D*_*y*_ = 0 nm; *M*_*cy*_ = 0, *M*_*cz*_ = 0. The optical torques of *x* component in the left-hand and right-hand side vortices have opposite signs. It means that the transverse spins in *x* direction of the NP trapped in the two different vortices are with the opposite helicities. Of interest is that the helicities of the spin and spiral orbit of the NP in *x* direction are opposite for individual optical vortex. If NP is trapped by the left-hand side optical vortex, for example, the NP performs a clockwise (CW) spin, whereas moves along a counter-clockwise (CCW) spiral orbit. In contrast, the NP trapped by the right-hand side vortex performs CCW spin and CW spiral orbit. The spiral motion coupled with spinning manifests the spin-momentum locking, particularly transverse SOI, even though the system is irradiated by a LP light. The significant SOI is contributed to the strongly twisted EM field in the near field of plasmonic nanostructure.

### Step-like motion caused by the growth and decline of optical vortices

**Figure 3:**
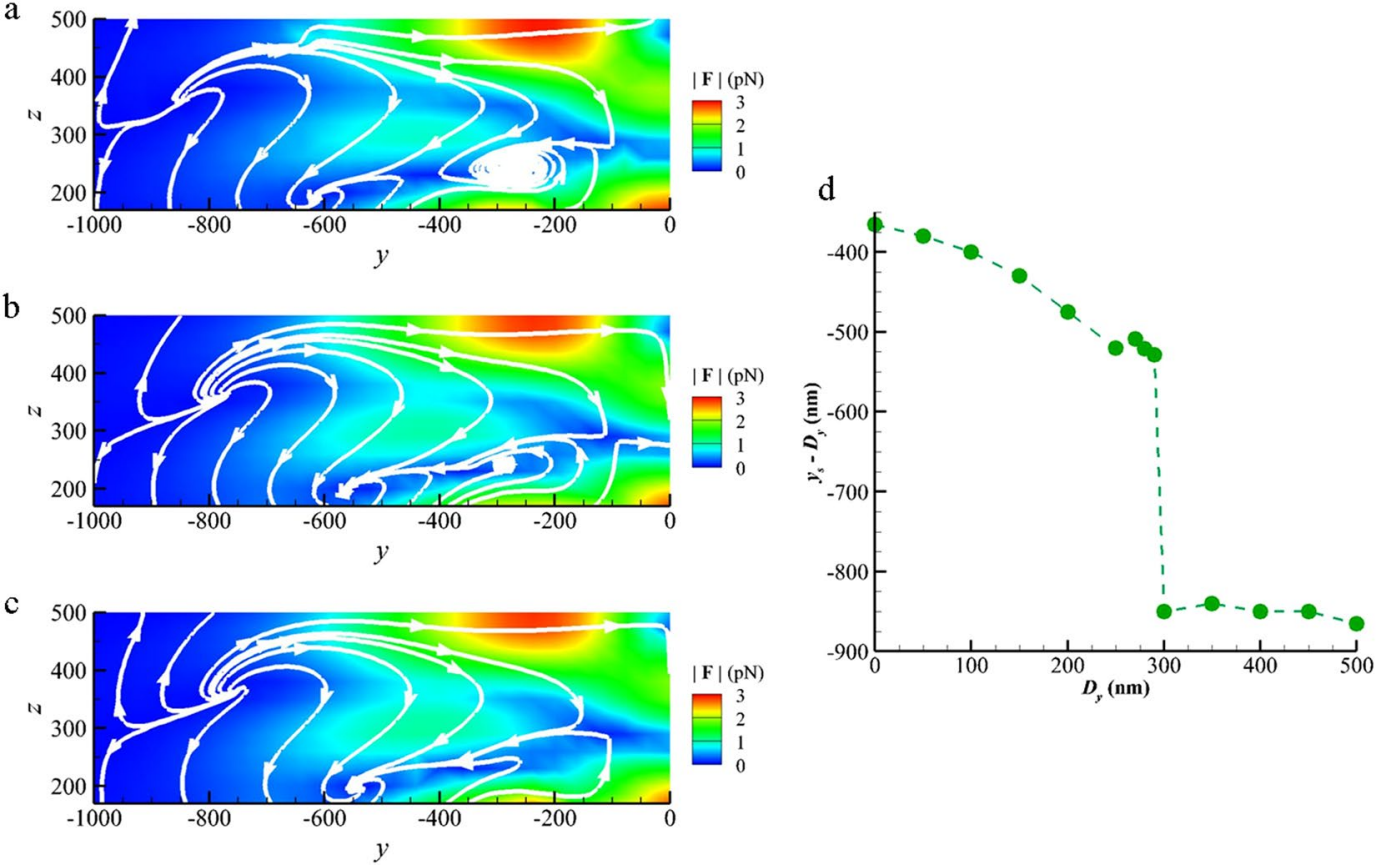
Step-like motion of NP along with the growth and decline of optical vortices. The streamline maps of the optical force field at the left-hand side optical vortex for (**a**) *D*_*y*_ = 250 *nm*, (**b**) *D*_*y*_ = 290 *nm* and (**c**) *D*_*y*_ = 300 *nm*. (**d**) The relative movement of stagnation point (*y*_*s*_ *− D*_*y*_) versus *D*_*y*_. The step-like motion of NP is caused by the growth of the new optical vortex and the decline of the old one.

When the 1D dimer array continuously moves along the array axis in the positive *y* direction away from the optical axis of Gaussian beam (i.e. *D*_*y*_ increases), the NP is trapped at the stagnation point in an optical vortex corresponding to a specific dimer and moves with the array to the right initially. If the NP is trapped by the left-hand side optical vortex initially at the stagnation point of (0, −361 *nm*, 207 *nm*) for the case of *D*_*y*_ = 0 *nm*, it will move with the trapping dimer centered at *y* = −500 *nm* toward the right first. If *D*_*y*_ is within a threshold, the trapping force of the optical vortex is dominant over the gradient force of Gaussian beam; the NP moves and follows the trapping dimer. As the moving distance of the dimer array increases, the trapped NP becomes closer to the Gaussian beam center and another new optical vortex is growing up in the vicinity of the next dimer, far from the Gaussian beam (Supplementary Video). Figure 3a indicates that there are two optical vortices with intact structure for *D*_*y*_ = 250 *nm*; the stagnation point of the trapping vortex is at *y*_*s*_ = −270 *nm* (the corresponding dimer centered at *y* = −250 *nm*) and that of a new one growing up at *y*_*s*_ = −620 *nm* (the corresponding dimer centered at *y* = −750 *nm*). As *D*_*y*_ continuously increases, the new vortex becomes stronger than the previous one, and then the trapped NP will be pushed out suddenly, from the previous stagnation point to the new one. For example, when *D*_*y*_ = 290 *nm* the new vortex with a center at *y*_*s*_ = −570 *nm* is firmed up whereas the previous one at *y*_*s*_ = −280 *nm* becomes loose, as shown in Fig. 3b. Moreover, Fig. 3b shows that there is a channel with nearly zero-force bridging the two vortices; it will become a pathway for the trapped NP leaping from the previous stagnation point to the new one as *D*_*y*_ increases. After *D*_*y*_ = 300 *nm*, the previous optical vortex completely disappears and the NP will be stably trapped by the new one at *y*_*s*_ = −550 *nm*, as shown in Fig. 3c. From these evidences, we can conclude that the leaping of the trapped NP almost occurs between *D*_*y*_ = 290 *nm* and 300 *nm*; the jump distance of the NP roughly is 290 *nm*, which is less than the lattice constant (500 *nm*). Through the growth and decline of optical vortices, a step-like motion of the NP relatively to the dimer array is caused, which is a nonlinear motion^15^. The step-like motion of the NP trapped by the left-hand side vortex is plotted in Fig. 3d. The optomechanical mechanism is complicated, involving the multiple forces in balance, e.g. the trapping force of dimer and the gradient force of Gaussian beam. On the other hand, if the NP is trapped by the right-hand side optical vortex initially at (0, 361 *nm*, 207 *nm*) for *D*_*y*_ = 0 *nm*, the same behavior is also found (see Figure s1 in Supplementary Information).

### Gap effect of dimer on optical vortex

**Figure 4:**
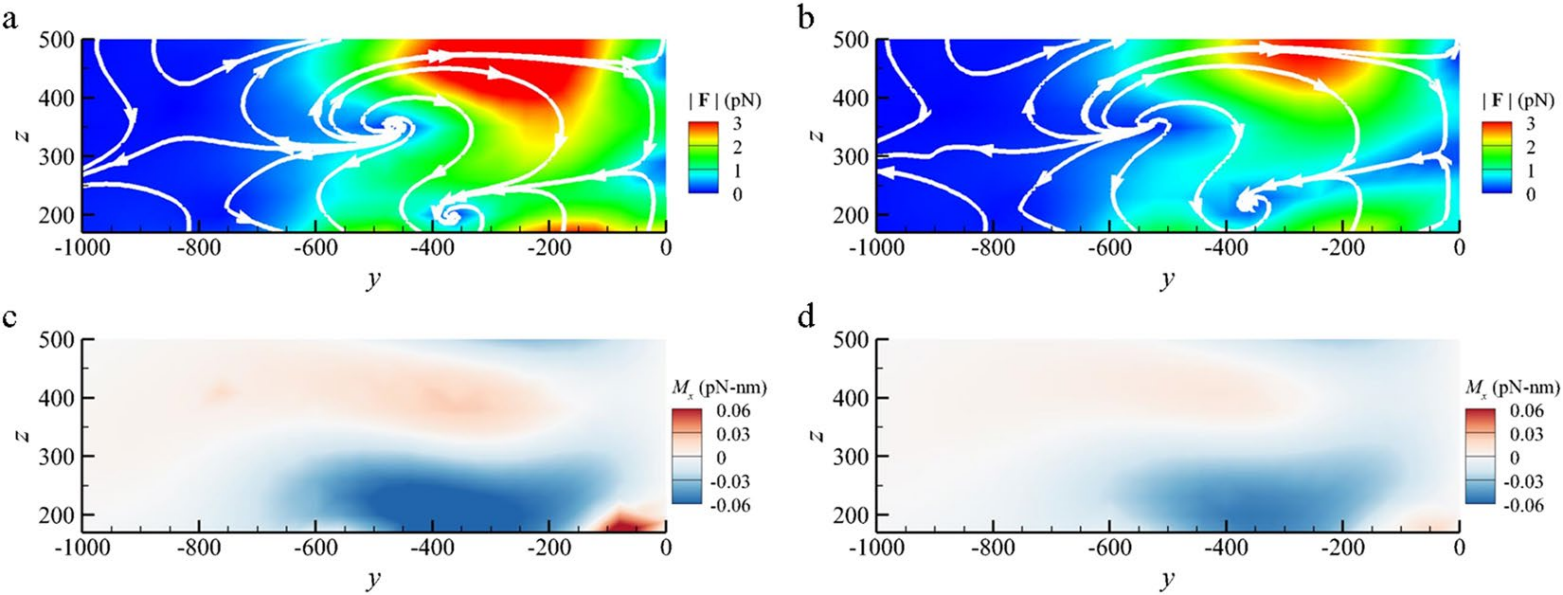
Gap effect of dimer on optical force and torque. (**a**) The streamline maps of the optical force field at the left-hand side optical vortex for *d* = 10 *nm* and (**b**) *d* = 50 *nm*. (**c**,**d**) The maps of optical spin torque field, *M*_*cx*_, for *d* = 10 *nm* and 50 *nm*, respectively. (*D*_*y*_ = 0 *nm*, *h*_*f*_ = 700 *nm*).

The dimer array, which is a metasurface nanoantennas structure, can be optimized for optical trapping. Since the gap zone of dimer is a hotspot of electric field, the gap effect of gold dimer on the optical vortex is considered a key factor. Figure 4a and b show the streamline maps of the optical force field for dimer arrays with different gaps (*d* = 10 *nm* and 50 *nm*) for *D*_*y*_ = 0 *nm*. The stiffness (spring constant) of the optical vortex is a tensor, which is defined as ${\boldsymbol{\kappa }}=-\,\nabla {\bf{F}}$ at the stagnation point. Comparing Fig. 4a and b with Fig. 2a of *d* = 30 *nm*, we found that the gradient of optical force in the vicinity of stagnation point is increased as the gap is reduced. It means that the trapping ability (stiffness) of the plasmonic dimer array on a probing NP is raised as we minimize the gap to enhance the plasmonic coupling in dimer. In addition, Fig. 4c and d show that the transverse optical spin torque (*M*_*cx*_) upon the trapped NP is also increased as the gap is reduced; the transverse SOI becomes more significant for a dimer array with smaller gap. To appropriately tailor the plasmonic nanostructure is helpful for enhancing the optical trapping and SOI. Nevertheless, the positions of the corresponding stagnation points of different-gap dimer arrays are almost the same.

### Off-focal plane effect on optical vortex

**Figure 5:**
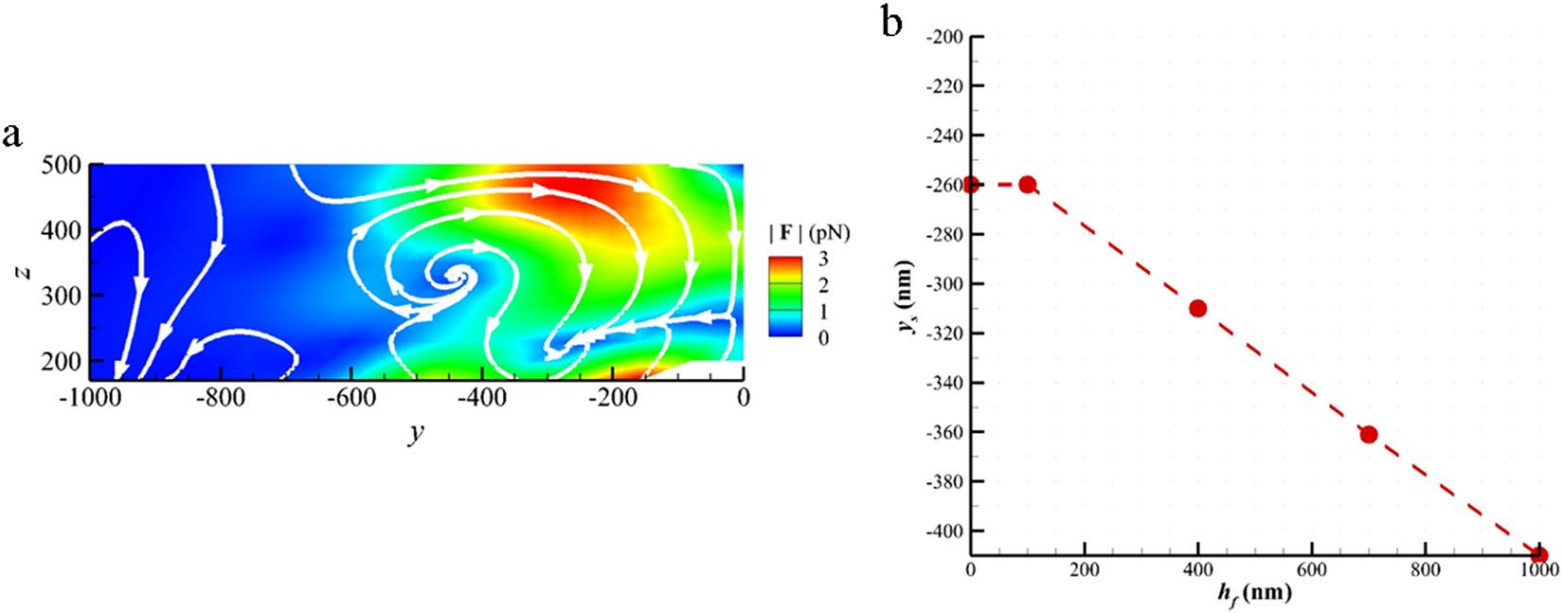
Focal-plane effect on optical vortex. (**a**) The streamline map of the optical force field at the left-hand side optical vortex for *h*_*f*_ = 100 *nm*. (**b**) The *y*_*s*_ of stagnation point versus *h*_*f*_. The trapping dimer is at *y* = −500 *nm*. (*d* = 30 *nm*, *D*_*y*_ = 0 *nm*).

We also investigate the off-focal plane effect on the optical vortex by altering the distance *h*_*f*_ (0, 100, 400, or 1000 *nm*) between the focal plane and the substrate at *z* = 0 for the case of *D*_*y*_ = 0. For example, for *h*_*f*_ = 100 *nm*, the stagnation points are at ${{\bf{x}}}_{s}=(0,\,\pm \,260\,nm,\,230\,nm)$, as shown in Fig. 5a. The other results are shown in Supplementary Information (Figure s2). For different *h*_*f*_, the optical vortices are still observed, but the location of the stagnation point changes. The relation of the *y*_*s*_ position of the stagnation point in the left-hand side optical vortex versus *h*_*f*_ is plotted in Fig. 5b. As *h*_*f*_ is reduced, the two optical vortices approach the central dimer. On the contrary, the two optical vortices approach the right-hand side and left-hand side dimers as *h*_*f*_ is increased. These results demonstrate that the precise positioning of optical vortex trapping can be easily implemented by adjusting *h*_*f*_ of the focal plane.

### Other factors on optical trapping & optical vortex

Because the existence of a dielectric NP disturbs the near field of plasmonic nanostructure, the size of the trapped NP affects the behavior of optical vortex. For example, the results of NP with a radius of 50 *nm* or 150 *nm* are shown in Supplementary Information (Figure s3), where the gap of dimer is 30 *nm*. We found that the optical force is roughly proportional to *a*^3^, the volume of NP, and the position of the stagnation point nearly does not change with the size. The finding is in accordance with the dipole approximation^13^. For the same irradiance the terminal speed of a larger NP is higher than that of a smaller one. Since the amplitudes of the optical force and torque are linearly proportional to the intensity of light, they are adjustable on demand to overcome the Brownian motion from the medium.

On the other hand, we can increase the numerical aperture of objective by tuning the iris to decrease the waist of Gaussian beam. For the 1064-*nm* Gaussian beam with a smaller waist, say *w*_*0*_ = 400 *nm*, the streamline map is shown in Figure s4 of Supplementary Information. The stagnation points for the probing NP are at (0, ±370 *nm*, 206 *nm*), where *h*_*f*_ = 700 *nm*, *d* = 30 *nm* and *D*_*y*_ = 0. The minimum gap between the dimer and the NP is 91 *nm*; the trapping mode is still non-contact. In comparison with the case of *w*_*0*_ = 500 *nm*, the horizontal position of stagnation points of *w*_*0*_ = 400 *nm* are more close to the corresponding dimers at *y* = ±500 *nm*.

If the polarization of light is perpendicular to the dimer (i.e. *y*-polarization), the non-contact mode (optical vortex) and the contact mode of dimer array’s trapping disappear, as shown in Supplementary Information (Figure s5). For this case, the Gaussian beam dominates the trapping. This illustrates the polarization-dependent optical vortex trapping. In addition, the size effect of dimer on optical vortex is also discussed in Supplementary Information (Figure s6). On the other hand, the intensity field ${\Vert {\bf{E}}\Vert }^{2}$ map in the *yz* plane of *x* = 0 is also provided in Supplementary Information (Figure s7). However, it is difficult to observe the optical vortex trapping from the intensity field. In fact, not only a dimer array but also a single dimer can produce the optical vortex trapping. The detailed description of the optomechanical behavior of a single dimer is provided in Supplementary Information (Figure s8).

## Discussion

The 3D optical vortex trapping of a 1D plasmonic dimer array upon a probing NP as irradiated by a LP Gaussian beam was revealed and identified numerically. We used the streamline map of optical force field representing the trajectory of NP’s quasi-static motion to visualize the contact and non-contact modes for trapping. In particular, for the non-contact mode we found that two optical vortices with opposite helicities are formed in the vicinity of the left-hand and right-hand side dimers of LP Gaussian beam. The dielectric NP can be stably trapped at a stagnation point in either optical vortex, and floats in the medium without touching the corresponding dimer. The optical vortex generates a 3D spiral trajectory of the NP toward the stagnation point, an orbital motion combined with a centripetal motion. In addition, we found that the probing NP can obtain transverse SAM due to the optical torque. No matter at the focal or off-focal plane of Gaussian beam, the optical vortex can be generated; the location of optical vortex can be controlled by the height of focal plane. The spirally orbital motion coupled with spinning of the probing NP is the result of spin-momentum locking, even though the system is irradiated by LP light and the NP is optical isotropic. The transverse SOI is manifested from the opposite helicities of the spin and spiral orbit of the probing NP. The phenomenon is similar to that a light-driven NP simultaneously obtains the linear momentum and transverse SAM in an evanescent or surface plasmon polarition wave^30–36^. Through the growth and decline of optical vortices, we quantitatively explained the step-like motion of the trapped NP as the 1D dimer array continuously moves away from the optical axis of LP Gaussian beam. Moreover, the trapping ability of a plasmonic dimer array on a probing NP and the transverse SOI can be enhanced by reducing the dimer’s gap. From the aspect of optical potential, the optical force can be expressed by ${\bf{F}}\approx -\,\nabla U$^16^. The stagnation point is the zone of the optical potential well, which is with zero force. However, we found that the optical force field in the vicinity of optical vortex is non-conservative;$\nabla \times {\bf{F}}\ne {\bf{0}}$^45,46^. The non-conservative behavior of optical vortex is worth exploring further.

This optical vortex is analogous to a fluidic one. When a stream flow encounters barriers, several vortices near the barriers could be generated. Additionally, the stagnation point of optical vortex is similar to the eye of typhoon, having strong trapping ability. The Maxwell’s stress tensor **T** is the linear momentum flux of EM field, which is equivalent to the linear momentum flux of fluid, $\rho \,{\bf{v}}\otimes {\bf{v}}$; *ρ* is the density and **v** the velocity of fluid. In general, linear momentum flux is a second-order tensor. Therefore, we used the Maxwell’s stress tensor, rather than the Poynting vector (energy flux), to exhibit the optical force and torque.

Unlike the conventional optical tweezers with a wavelength-sized trapping zone, a plasmonic dimer array interacting with LP Gaussian beam can induce 3D optical vortex trapping to confine a dielectric NP at aN stagnation point in the near field of the nanostructure with a subwavelength-sized zone. In addition, the SOI becomes profound in the optical vortex zone. Besides the non-contact mode (optical vortex trapping), the contact mode is also induced by this plasmonic nanostructure. These new findings pave the way for the applications of using plasmonic metasurface to enhance optomechanics on subwavelength manipulation in lab-on-a-chip devices and nanofluidics.

## Methods

We simplify the model for simulation without considering the substrate effect. The configuration of 1D gold dimer array is shown in Fig. 1. The cross section plane of the array is at *z* = 0. The array is irradiated by a normally incident LP Gaussian beam of 1064 *nm*. The relative permittivity of gold provided in ref.^52^ is used for simulation; *ε*_*r*_ = (−48.45, 3.6). The polarization of beam is parallel to the dimer center line, and perpendicular to the array line. The MMP method was utilized to numerically analyze the EM field of Gaussian beam interacting with a dimer array and a probing NP, where five or six dimers were used for simulation. All the interfaces of these dimers and the polystyrene NP are discretized for the calculation of the coupled EM field induced by the interaction of the dimer array and the NP with Gaussian beam. In this paper, the harmonic time factor is ${e}^{-i\omega t}$, where $\omega$ is the angular frequency and $i=\sqrt{-1}$. In terms of the total field (**E**, **H**) of the exterior field, the time averaged Poynting vector **S**, which is the flux of energy, is given by

1 $${\bf{S}}=\frac{1}{2}\Re ({\bf{E}}\times \bar{{\bf{H}}}).$$

where $\Re$ represents the real part and the over bar the complex conjugate. The Maxwell’s stress tensor **T** is the time-averaged linear momentum flux,

2 $${\bf{T}}=\frac{1}{2}\Re \{\varepsilon {\bf{E}}\otimes \bar{{\bf{E}}}+\mu {\bf{H}}\otimes \bar{{\bf{H}}}-\frac{1}{2}(\varepsilon {\bf{E}}\cdot \bar{{\bf{E}}}+\mu {\bf{H}}\cdot \bar{{\bf{H}}}){\bf{I}}\},$$

which is a second-order tensor. In Eq. (2), **I** is a unit second-order tensor of 3 by 3. Here $\varepsilon$ and $\mu$ are the permittivity and permeability of the medium. The optical force exerted on the polystyrene NP, in terms of **T**, is given by

3 $${\bf{F}}({{\bf{x}}}_{c})={\int }_{S}{\bf{T}}\cdot {\bf{n}}{\rm{d}}s,$$

where *S* is the surface of the probing NP, and **x**_*c*_ is the position vector of the mass center of the NP. Moreover, the optical torque is expressed by^49,50^

4 $${\bf{M}}({{\bf{x}}}_{c})={\int }_{S}{\bf{r}}\times {\bf{T}}\cdot {\bf{n}}{\rm{d}}s,$$

where ${\bf{r}}\times {\bf{T}}$ is the angular momentum flux and **r** is the relative position vector of a point **x** on the surface *S w.r.t*. a given reference point **x**_*o*_; ${\bf{r}}={\bf{x}}-{{\bf{x}}}_{o}$. The **r** can be expressed by ${\bf{r}}={\bf{r}}{\boldsymbol{^{\prime} }}+{{\bf{r}}}_{c}$, where ${{\bf{r}}}_{c}={{\bf{x}}}_{c}-{{\bf{x}}}_{o}$ and ${\bf{r}}{\boldsymbol{^{\prime} }}={\bf{x}}-{{\bf{x}}}_{c}$. Hence, the optical torque can be decomposed into

5 $${\bf{M}}={\int }_{S}{\bf{r}}{\boldsymbol{^{\prime} }}\times {\bf{T}}\cdot {\bf{n}}{\rm{d}}s+{{\bf{r}}}_{c}\times {\bf{F}}.$$

The first term of the right-hand side of Eq. (5) is the optical spin torque ${{\bf{M}}}_{c}$ for the NP’s spin, where ${\bf{r}}{\boldsymbol{^{\prime} }}\times {\bf{T}}$ is the SAM flux. The second term of Eq. (5) is the optical orbital torque for the orbital motion of the NP; ${{\bf{r}}}_{c}\times {\bf{F}}$. The former changes the SAM of the NP, and the latter the OAM, which is related to the optical force. In this paper, we choose the stagnation point of the optical vortex as the reference point; **x**_*o*_** = x**_*s*_.

## Electronic supplementary material

Supplementary Information 1

 Supplementary Information 2
