# Supplementary material for: 3D Optical Vortex Trapping of Plasmonic Nanostructure
Source: Sci Rep. 2018 Aug 23;8:12673. doi: 10.1038/s41598-018-30948-y (PMC6107535; doi:10.1038/s41598-018-30948-y)
Supplement: Supplementary file 1 — Supplementary Information 1 [file 41598_2018_30948_MOESM1_ESM.docx]

**SUPPLEMENTARY INFORMATION**

**3D Optical Vortex Trapping of Plasmonic Nanostructure**

Jiunn-Woei Liaw^1,2,3,4*^, Chiao-Wei Chien^5^, Kun-Chi Liu^5^, Yun-Cheng Ku^5^, Mao-Kuen Kuo^5*^

^1^Department of Mechanical Engineering, Chang Gung University, Taiwan

^2^Institute for Radiological Research, Chang Gung University and Chang Gung Memorial Hospital, Taiwan

^3^Center for Advanced Molecular Imaging and Translation, Chang Gung Memorial Hospital, Linkou, Taiwan

^4^Department of Mechanical Engineering, Ming Chi University of Technology, Taiwan

^5^Institute of Applied Mechanics, National Taiwan University, Taiwan

E-mail address: [markliaw@mail.cgu.edu.tw](mailto:markliaw@mail.cgu.edu.tw), [mkkuo@ntu.edu.tw](mailto:mkkuo@ntu.edu.tw)

In this paper, we revisited the optical-vortex trapping of a 1D gold dimer array on a polystyrene nanoparticle (NP) irradiated by a normally incident linearly polarized (LP) Gaussian beam; the previous experimental measurement has been reported in ref. 1. The surrounding medium is oil. The polarization of Gaussian beam is parallel to the dimer center line (*x*-polarization), and perpendicular to 1D dimer array line (*y* direction). The radius of each gold NP of dimer is 65 nm, and the lattice constant between dimers is 500 nm. The gap between dimer is denoted by *d*, and the radius of polystyrene NP is denoted by *a*. The distance between the Gaussian beam and the central dimer is denoted by *D_y_*. The height of the focal plane of Gaussian beam above the central cross section of dimer (substrate) is denoted by *h_f_*, and the waist by *w_0_*. Throughout this paper, the wavelength of Gaussian beam is *λ*= 1064 nm. We used multiple multipole (MMP) method for the simulation of EM field [2-5]. The optical force and torque, in terms of Maxwell’s stress tensor, exerted on the polystyrene NP were calculated [4, 5]. All the interfaces of these dimers and the polystyrene NP are discretized for the calculation of the coupled EM field. The refractive indexes of polystyrene NP and oil are 1.6 and 1.5, respectively.

**Supplemental Results and Discussion**

1. The step-like motion of NP trapped by the right-hand side optical vortex

If the NP is trapped by the right-hand side optical vortex initially at (0, 361 *nm*, 207 *nm*) for *D_y_* = 0 *nm*, it will follow the trapping dimer as the dimer array move to the right. As *D_y_* increases and is within a threshold, the trapping force of the optical vortex is dominant over the gradient force of Gaussian beam; the NP moves along with the trapping dimer. For *D_y_*= 250 *nm* (shown in Figure s1a), the structure of the previous optical vortex still exists and its stagnation point moves to *y_s_* = 610 *nm* (the corresponding dimer at *y*= 750 *nm*), while another new optical vortex with a stagnation point at *y_s_* = 260 *nm* (the corresponding dimer at *y*= 250 *nm*) is just growing up in the vicinity of the next dimer, closer to the Gaussian beam. As *D_y_* increases, the new optical vortex becomes stronger than the previous one. Consequently, the trapped NP will be drawn back, from the previous optical vortex to the new one. The jumping of the tapped NP may happen during *D_y_* = 250 *nm* to *D_y_*= 300 *nm*. The jump distance of the NP roughly is 350 *nm*, less than the lattice constant of array (500 *nm*). Through the growth and decline of optical vortices, the NP performs a step-like motion with respect to the dimer array as the array continuously moves to the right. Figure s1b show the result of *D_y_* = 350 *nm*. The new optical vortex traps the NP at the stagnation point of *y_s_* = 270 *nm* (the corresponding dimer at *y*= 350 *nm*), whereas the previous one completely disappears. The same result of *D_y_*= 400 *nm* is shown in Figure 1sc, where the stagnation point is at *y_s_* = 300 *nm*.


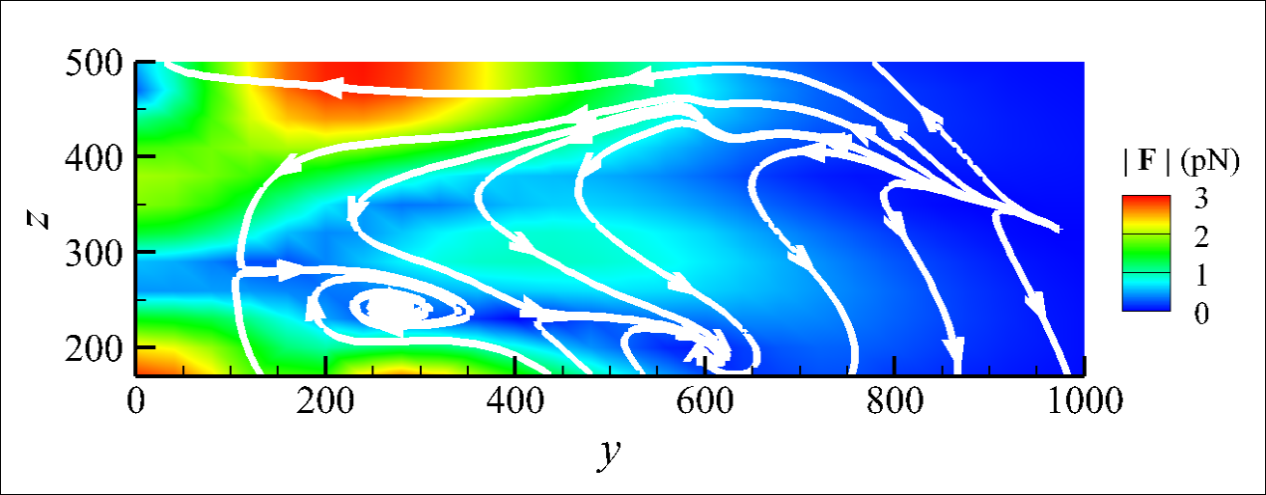


(a)


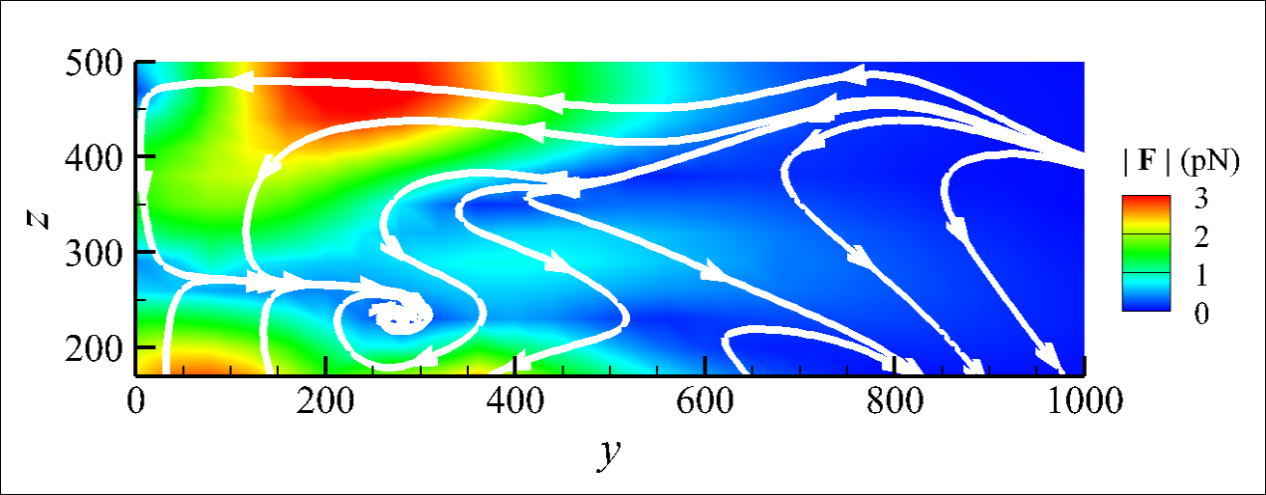


(b)


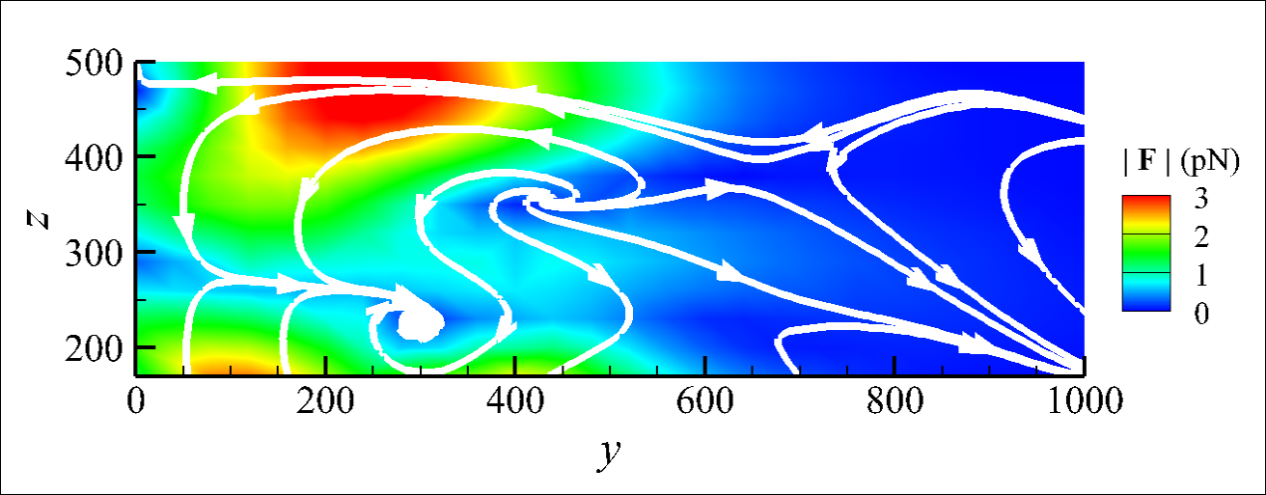


(c)

**Figure s1.** The streamline maps of the right-hand side optical force field in the *yz* plane of *x*= 0 for (a) *D_y_* = 250 *nm*, (b) *D_y_*= 350 *nm* and (c) *D_y_*= 400 *nm*. (*a*= 300 *nm*, *d*= 30 *nm*, *h_f_* = 700 *nm*, *w_0_* = 500 *nm*)

1. Effect of focal plane

We also investigated the focal-plane effect on the optical vortex by changing the distance (*h_f_* = 0, 400 *nm*, 1000 *nm*) between the focal plane and the substrate at *z*= 0 for the case of *D_y_*= 0. The streamline maps of the optical force for the left-hand side optical vortex are shown in Figure s2 for different *h_f_* (0, 400 *nm*, 1000 *nm*). The optical vortices are still observed, but the location of the stagnation point changes. The stagnation points of the cases of *h_f_* = 0, 400 *nm*, 1000 *nm* are at *y_s_*= -260 *nm*, -300 *nm* and -400 *nm*, respectively. These results imply that the optical vortices approach the central dimer as *h_f_* is reduced.


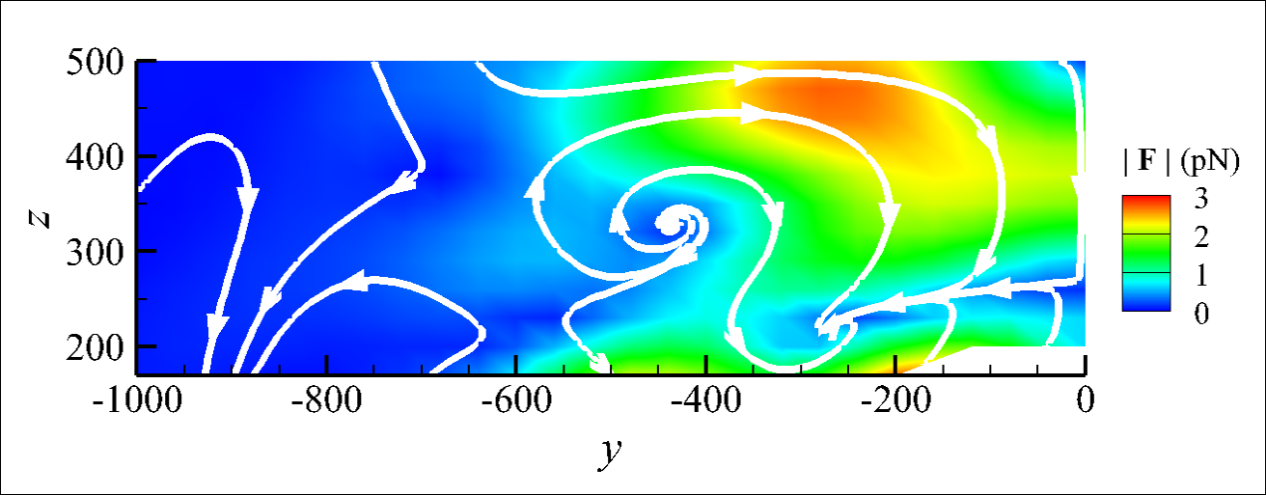
(a)


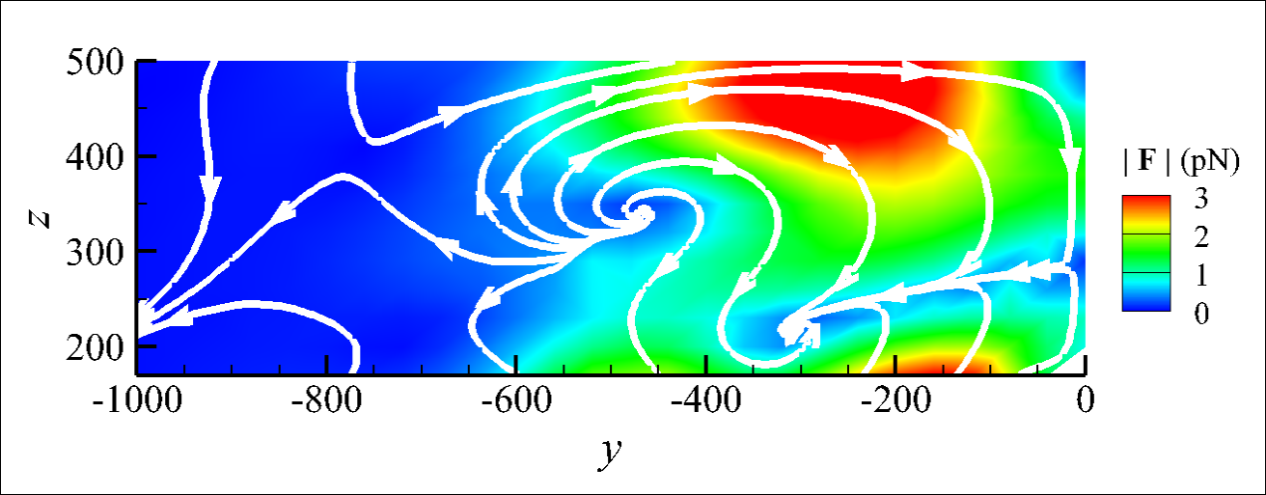


(b)


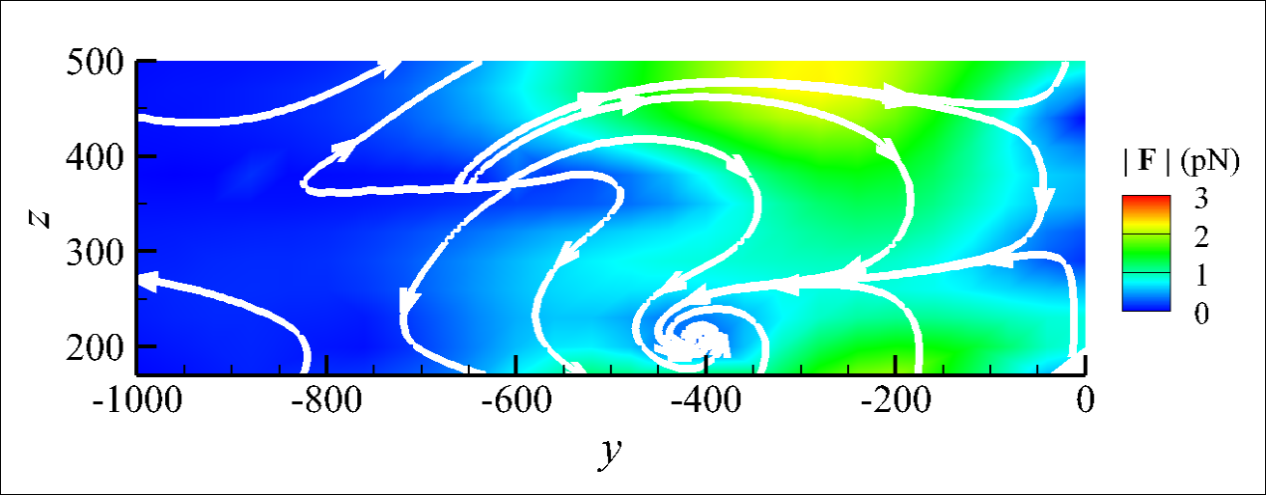


(c)

**Figure s2.** The streamline maps of the left-hand side optical force field in the *yz* plane of *x*= 0 for different *h_f_* of (a) 0 *nm*, (b) 400 *nm* and (c) 1000 *nm*, where *a*= 100 *nm*, *d*= 30 *nm*, *D_y_* = 0 and *w_0_* = 500 *nm*.

1. Size effect of probing NP on optical vortex

The results of NP with different radius of *a*= 50 *nm* and 150 *nm* are shown in Figure s3a and c, where the gap of dimer is 30 *nm*. Figure s3b is the zoom-in plot of Figure s3a. In comparison with the result of Figure 2a (*a*= 100 *nm*), the positions of the stagnation point are almost the same; *y_s_* = 360 *nm*. However, the magnitude of the optical force is almost proportional to the volume of the trapped NP; *a*^3^.


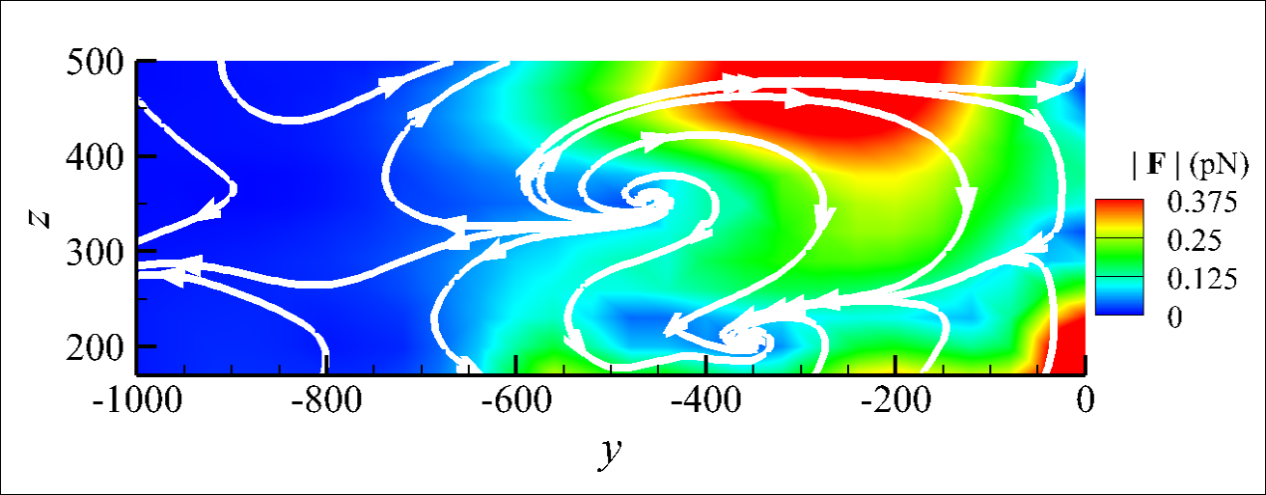


(a)


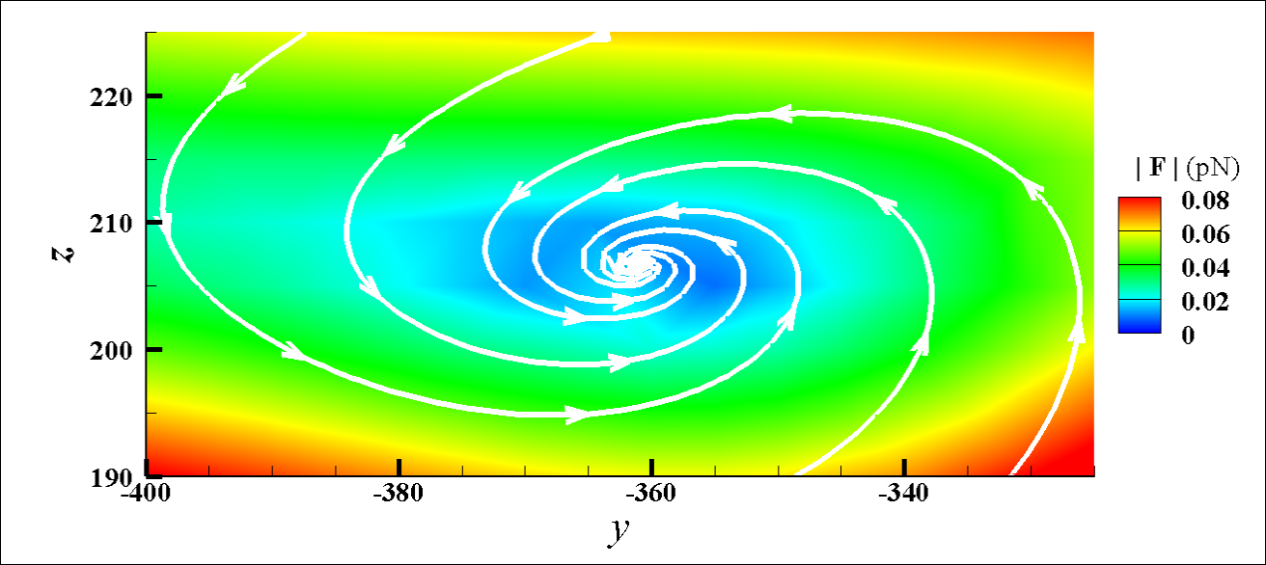


(b)


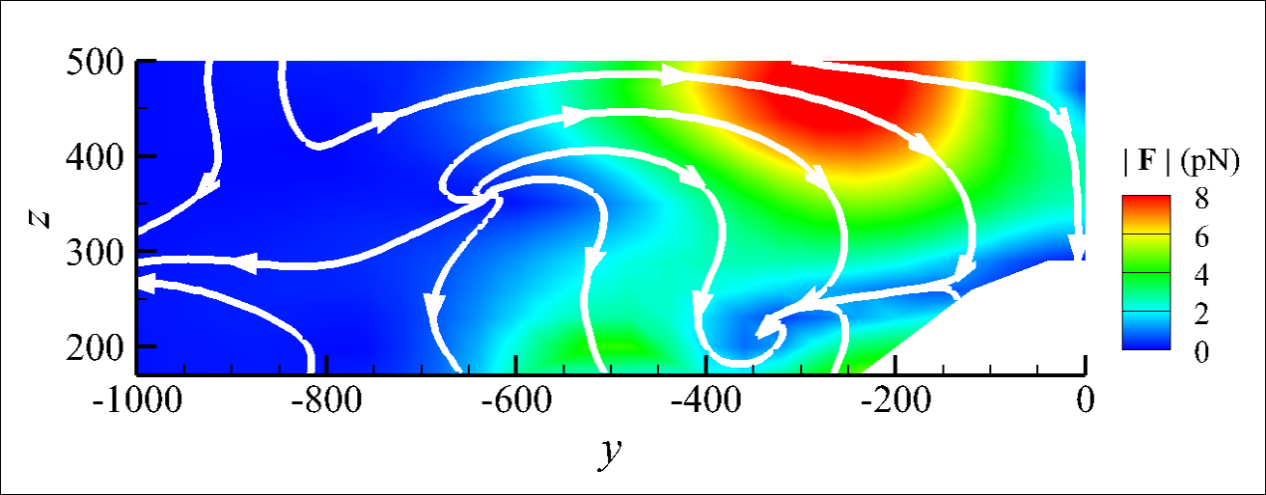
(c)

**Figure s3.** The streamline maps of the left-hand side optical force field for different sized probing NP of (a) *a*= 50 *nm* and (c) *a*= 150 *nm*; (b) is the zoom-in plot of (a) at the optical vortex. (*h_f_* = 700 *nm*, *d*= 30 *nm*, *w_0_* = 500 *nm*, *D_y_* = 0)

1. Waist of Gaussian beam effect

The streamline map the left-hand side optical force field of *w_0_* = 400 *nm* is shown in Figure s4, where *h_f_* = 700 *nm* and *D_y_*= 0. The stagnation point of the probing NP are at (0, -370 *nm*, 206 *nm*) for *w_0_* = 400 *nm*, whereas those points for *w_0_* = 500 *nm* are at (0, -361 *nm*, 207 *nm*). For the former the gap between the dimer and the NP is 91 *nm*, and that of the latter 97 nm. This is to say that the location of optical vortex is closer to the corresponding dimer as the waist of Gaussian beam is reduced.


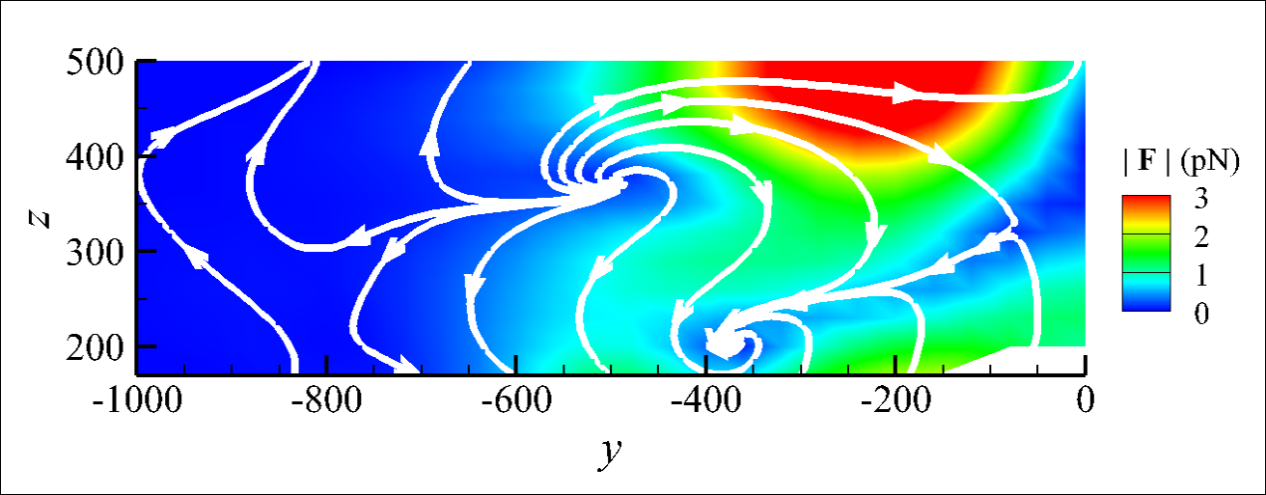


**Figure s4.** The streamline map of the left-hand side optical force field for *w_0_* = 400 *nm*, where *a*= 100 *nm*, *d*= 30 *nm*, *h_f_* = 700 *nm* and *D_y_* = 0.

1. Polarization dependence

In this research, the optical vortex trapping is found to be induced by an *x*-polarized Gaussian beam, parallel to the dimer orientation. If the polarization of light is perpendicular to the dimer (i.e. *y*-polarization), the polystyrene NP is also trapped to float at a stagnation point in the optical axis of Gaussian beam above the central dimer. However, the optical vortex (non-contact mode) and the contact mode of dimer array’s trapping disappear, as shown in Figure s5. For this case, the trapping of Gaussian beam is dominant over that of the dimer array. It means that the phenomenon of optical vortex trapping is polarization-dependent.


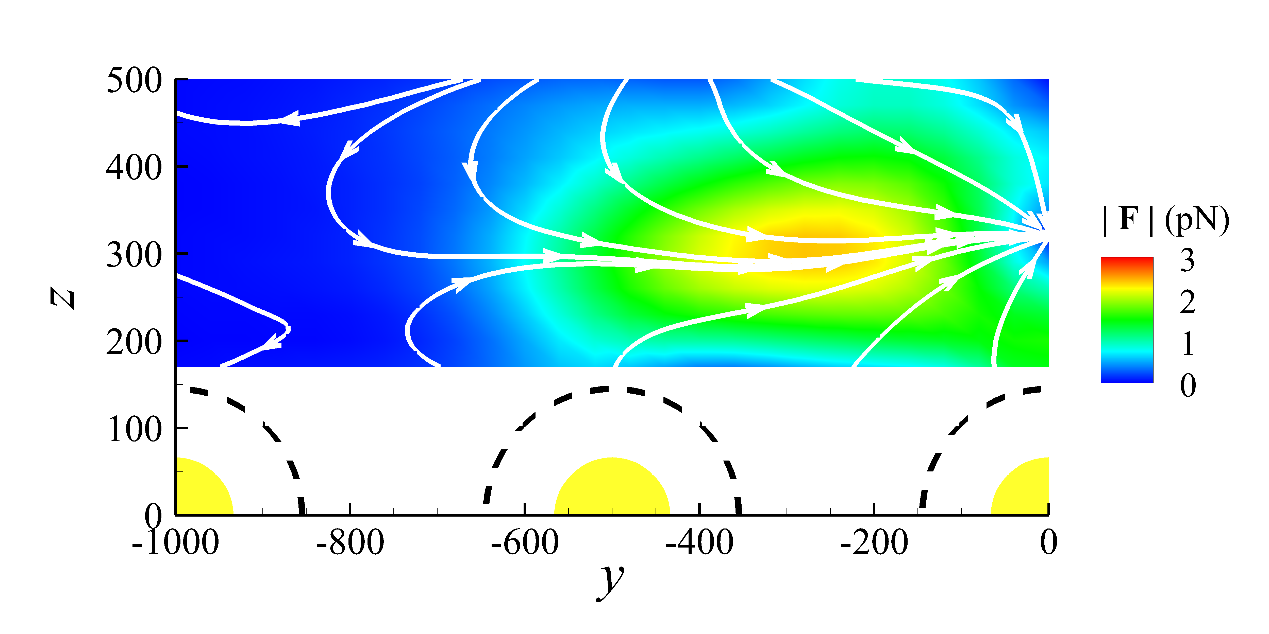


**Figure s5.** The streamline map of the left-hand side optical force field in the *yz* plane of *x*= 0 induced by a *y-*polarized light. (*h_f_* = 700 *nm*, *d* = 30 *nm*, *w_0_* = 500 *nm*, *D_y_* = 0, *a* = 100 *nm*)

1. Size effect of dimer on optical vortex

If we change the size of the gold NP of dimer, the behavior of optical vortex trapping is also changed. For example, Figure s6 shows the streamline maps of optical force for a dimer array, where the dimer’s gap is 30 nm but with different radii of gold NP (50 nm or 100 nm). In comparison with the result (Figure 2a) of a dimer array with gold NP of 65-nm radius, the optical vortex of the former (50 nm) moves to above the center dimer. In contrast, for the latter (100 nm) the contact mode dominates the trapping, but the optical vortex of the non-contact mode disappears. These results illustrate that the trapping performance of the contact and non-contact modes of dimer array is dependent of the size of gold NP.


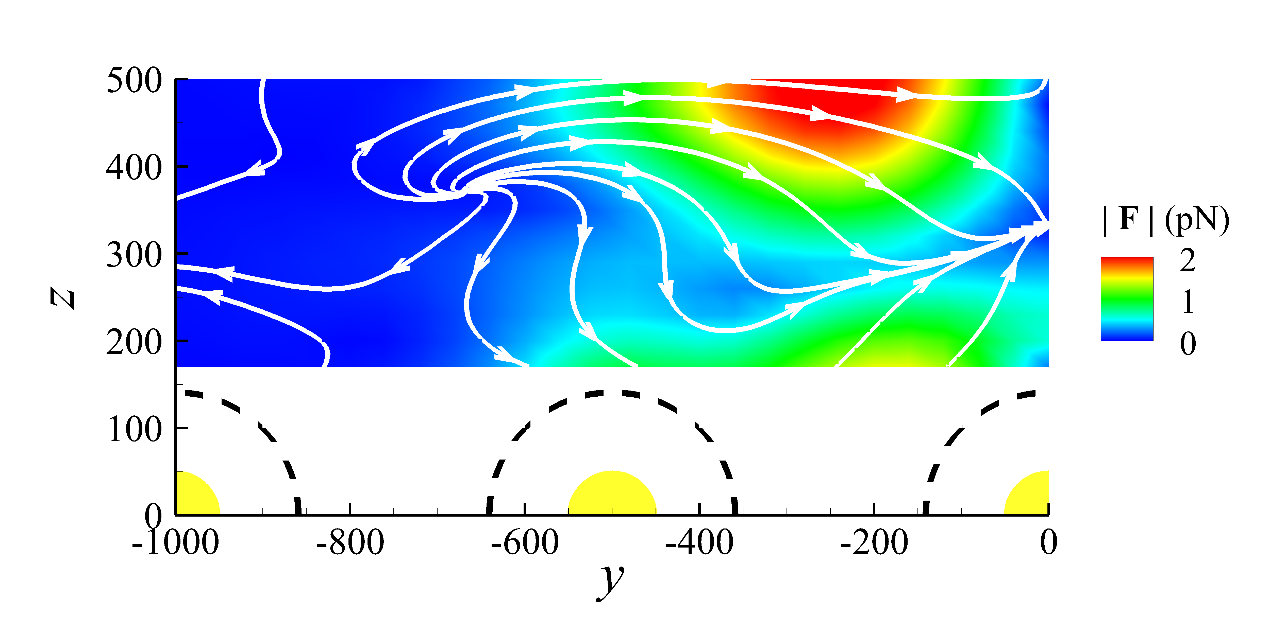


(a)


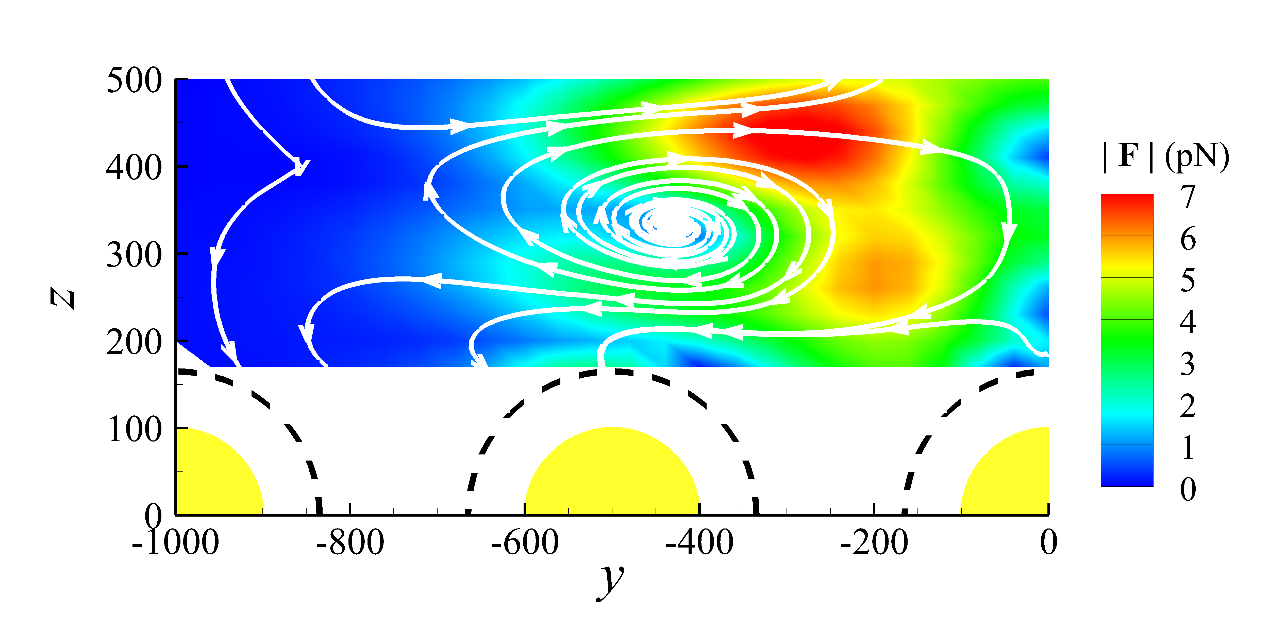


(b)

**Figure s6.** The streamline maps of the left-hand side optical force field of dimer array. The radius of each gold NP is (a) 50 nm and (b) 100 nm. (*h_f_* = 700 *nm*, *d* = 30 *nm*, *w_0_* = 500 *nm*, *D_y_* = 0, *a* = 100 *nm*)

1. Intensity field

The intensity field $\left\|\mathbf{E}\right\|^{2}$ map of the left-hand side in the *yz* plane of *x*= 0 is shown in Figure s7, where the polystyrene NP is trapped at the stagnation point (0, −361 *nm*, 207 *nm*). From this figure, it is difficult to observe the optical vortex trapping.


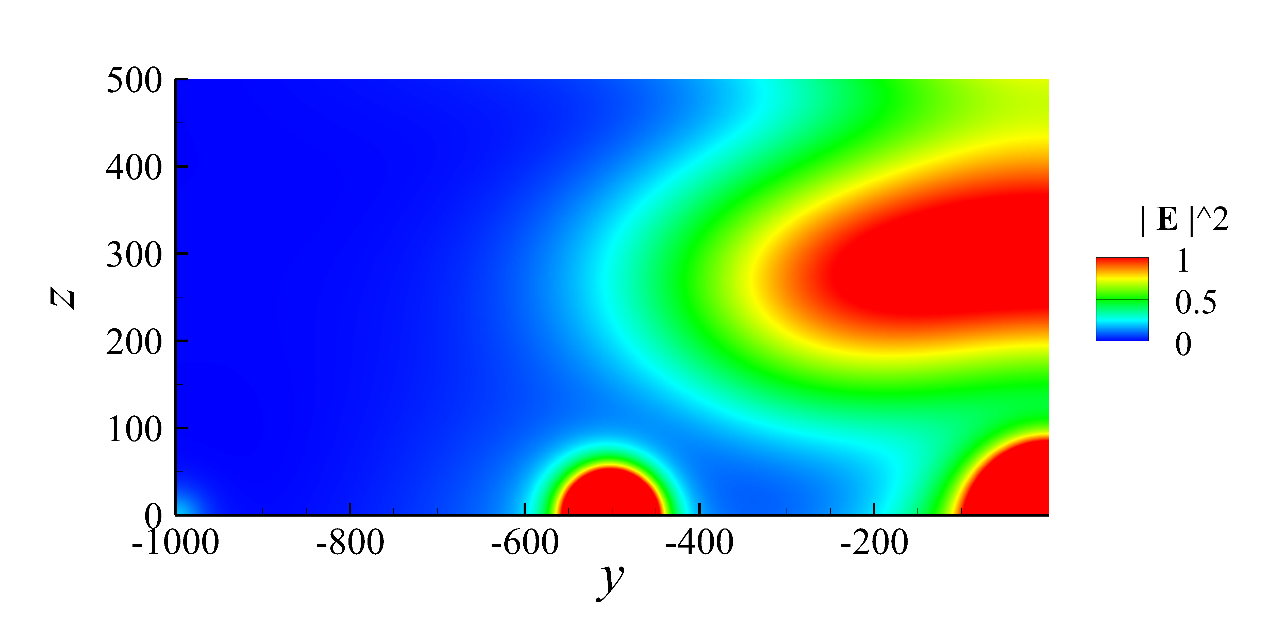


**Figure s7.** The intensity field $\left\|\mathbf{E}\right\|^{2}$ map of the left-hand side in the *yz* plane of *x*= 0, where the polystyrene NP is at the stagnation point (0, −361 *nm*, 207 *nm*). (*h_f_* = 700 *nm*, *d* = 30 *nm*, *w_0_* = 500 *nm*, *D_y_* = 0, *a* = 100 *nm*)

1. Single dimer

The 3D streamline map of the optical force field of a single dimer at *D_y_* = 0 upon a polystyrene NP is shown in Figure s8a, and the 2D streamline map at the *yz* plane of *x*= 0 in Figure s8b, where the radius of each gold NP is 65 nm. There are also two optical vortexes at the both sides of a single gold dimer. It demonstrates that the optical vortex trapping can also be found for a single gold dimer irradiated by *x*-polarized Gaussian beam.


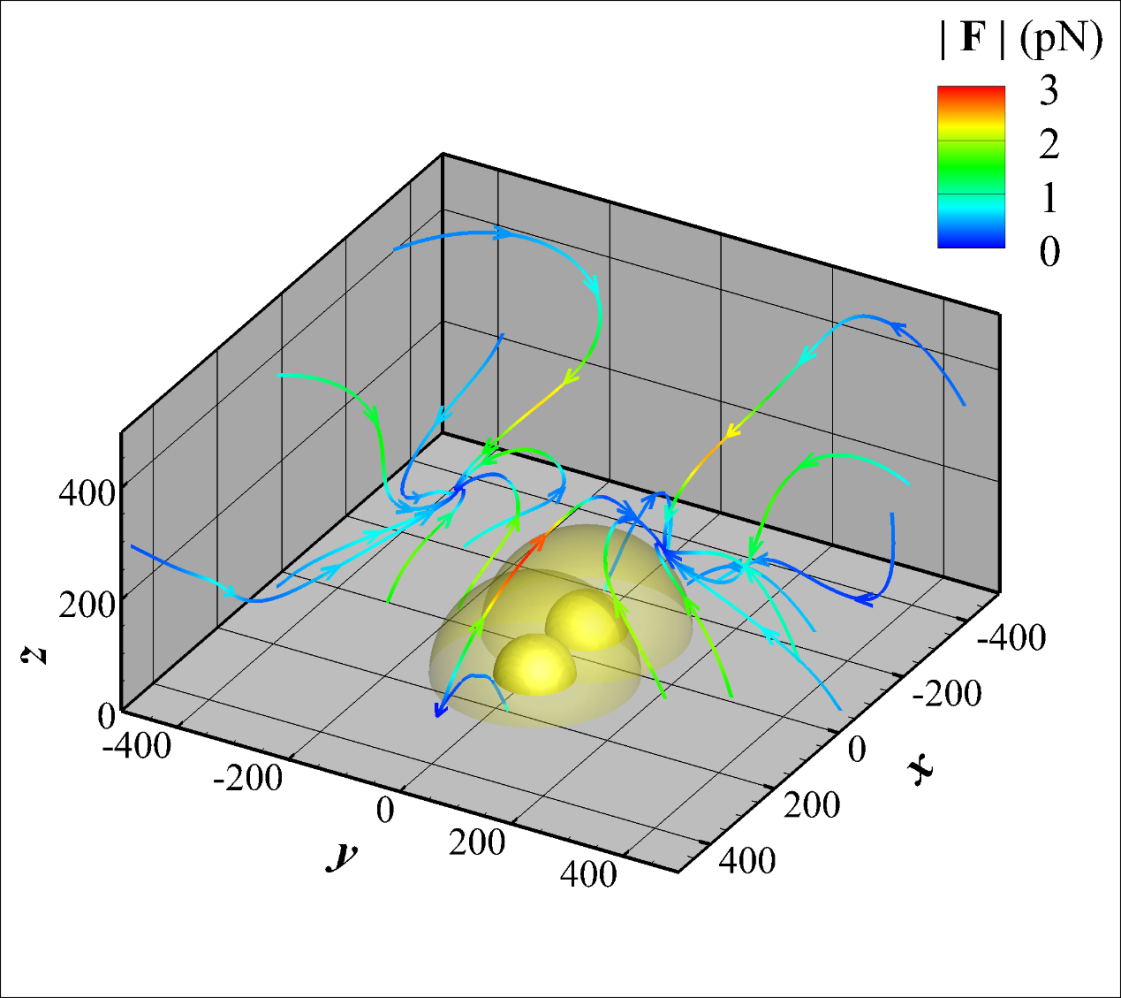


(a)


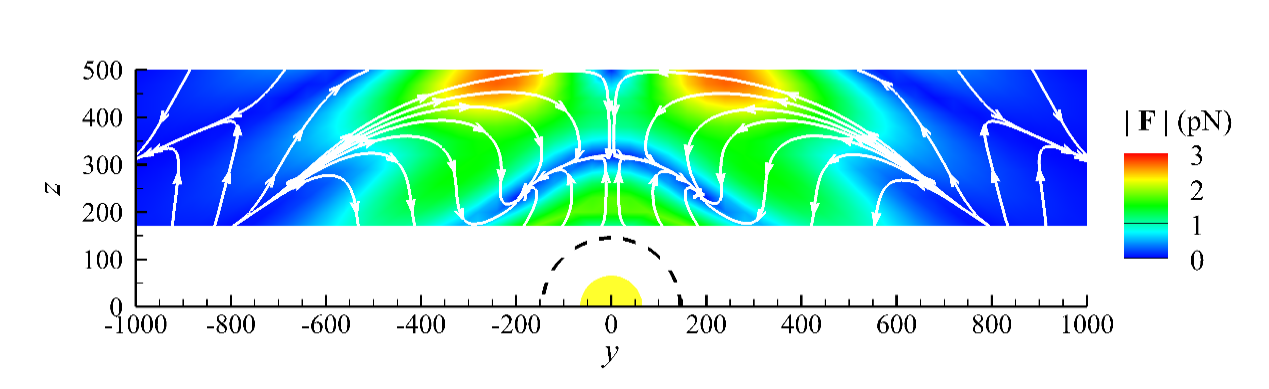


(b)

**Figure s8.** (a) The 3D streamline map of the optical force field of a single dimer at *D_y_* = 0 irradiated by *x*-polarized Gaussian beam. (b) The 2D streamline map at the *yz* plane of *x*= 0. (*h_f_* = 700 *nm*, *d* = 30 *nm*, *w_0_* = 500 *nm*, *D_y_* = 0, *a* = 100 *nm*)

**Reference**

1. Grigorenko, A. N., Roberts, N. W., Dickinson, M. R. & Zhang, Y. Nanometric optical tweezers based on nanostructured substrates. *Nat. Photon.* **2**, 365370 (2008).
2. Liaw, J.-W., Kuo, M. K. & Liao, C. N. Plasmon resonance of spherical and ellipsoidal nanoparticles. *J. Electromagn. Waves and Appl.* **19**(13), 1787−1794 (2005).
3. Liaw, J. W., Chen, C. S., Chen, J. H. & Kuo, M. K. Purcell effect of nanoshell dimer on single molecule’s fluorescence. *Optics Express* **17**(16), 13532−13540 (2009).
4. Liaw, J. W., Kuo, T. Y. & Kuo, M. K. Plasmon-mediated binding forces on gold or silver homodimer and heterodimer. *J. Quant. Spectrosc. Radiat. Transfer* **170**, 150−158 (2016).
5. Liaw, J. W., Lin, W. C. & Kuo, M. K. Wavelength-dependent plasmon-mediated coalescence of two gold nanorods. *Sci. Rep.* **7**, 46095 (2017).
