# Supplementary figures and images for: 3D Optical Vortex Trapping of Plasmonic Nanostructure
Source: Sci Rep. 2018 Aug 23;8:12673. doi: 10.1038/s41598-018-30948-y (PMC6107535; doi:10.1038/s41598-018-30948-y)

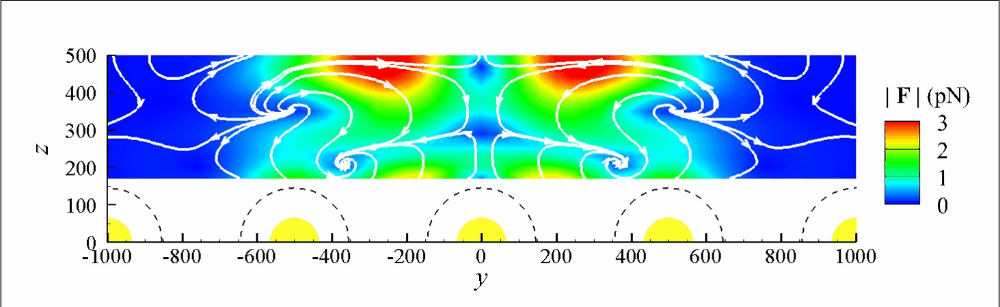

Supplement: Supplementary file 2 — Supplementary Information 2 [file 41598_2018_30948_MOESM2_ESM.gif]
